# Translating restrictive law into practice: An ethnographic exploration of the systemic processing of legally restricted health care access for asylum seekers in Germany

**DOI:** 10.1186/s12939-024-02251-y

**Published:** 2024-10-10

**Authors:** Sandra Ziegler, Kayvan Bozorgmehr

**Affiliations:** 1 Department of General Practice and Health Services Research, Section for Health Equity Studies & Migration, Heidelberg University Hospital, Im Neuenheimer Feld 130.3, Heidelberg, 69120 Germany; 2 Department of Population Medicine and Health Services Research, School of Public Health, University of Bielefeld, Universitätsstraße 25, Bielefeld, 33615 Germany

**Keywords:** Health care restrictions, Refugees, Asylum seekers, Health system, Cost coverage, Migration policies, Interpretation of law, Prioritization, Discretionary-decisions, Conflicting rationales, Street-level bureaucracy

## Abstract

**Background:**

Access to health services for asylum seekers is legally restricted in Germany. The law is subject to interpretation, therefore the chance of receiving care is not equally distributed among asylum seekers. What services are provided to whom is ultimately decided by health professionals and government employees. The respective prioritization processes and criteria are not transparent. We sought to understand how legal restrictions are translated into daily practices and how this affects the health system. We aimed to outline the complex process of cost coverage for health services for asylum seekers and provide insights into common decision-making criteria.

**Methods:**

We conducted an ethnographic exploration of routines in two outpatient clinics in two federal states over the course of three months, doing participant and non-participant observation. Additionally, we interviewed 21 professionals of health care and government organizations, and documented 110 applications for cost coverage of medical services and their outcome. In addition to qualitative data analysis and documentation, we apply a system-theoretical perspective to our findings.

**Results:**

To perform legal restrictions a cross-sectoral prioritization process of medical services has been implemented, involving health care and government institutions. This changes professional practices, responsibilities and (power) relations. Involved actors find themselves at the intersection of several, oftentimes conflicting priorities, since “doing it right” might be seen differently from a legal, medical, economic, or political perspective. The system-theoretical analysis reveals that while actors have to bring different rationales into workable arrangements this part of the medical system transforms, giving rise to a sub-system that incorporates migration political rationales.

**Conclusions:**

Health care restrictions for asylum seekers are implemented through an organizational linking of care provision and government administration, resulting in a bureaucratization of practice. Power structures at this intersection of health and migration policy, that are uncommon in other parts of the health system are thereby normalized. Outpatient clinics provide low-threshold access to health services, but paradoxically they may unintentionally stabilize health inequities, if prioritization criteria and power dynamics are not made transparent. Health professionals should openly reflect on conflicting rationales. Training, research and professional associations need to empower them to stay true to professional ethical principles and international conventions.

**Supplementary Information:**

The online version contains supplementary material available at 10.1186/s12939-024-02251-y.

## Background

### Health care access for asylum seekers in Europe: aims and structure of an exploration of the German case

People seeking international protection still face legal barriers to accessing health services upon arrival in many European countries [1–4]. While most countries provide some level of coverage, it often takes time for the access to healthcare to align with that of the general population. During this period, access may be restricted in a specific way (to compare regulations see: [1, 5, 6]). One obstacle is the requirement to reside in a country for a minimum period before becoming eligible for integration into its general healthcare system [7]. These regulations vary widely among European states and actual access can also differ regionally within these states. For example, in Austria, an electronic health card and/or insurance number can be issued after a few days, granting the same access as for other beneficiaries [8]. In contrast adult asylum seekers in France must wait three months [9], and in Germany the waiting period can be even longer. In a country where health insurance coverage is mandatory for citizens [10, 11], non-citizens seeking protection are only granted reduced rights for up to 36 months.

This article focusses on the challenges and systemic issues related to healthcare access for asylum seekers in Germany. It highlights legal barriers and bureaucratic complexities asylum seekers face, such as waiting periods and restricted access to healthcare services. It shows how legal healthcare restrictions are translated into practice, discussing the decentralized and often non-transparent nature of the decision-making processes under Germany's Asylum Seekers Benefits Act (ASBA). Combining an ethnographic empirical approach with theoretical reflections, the article analyses how these restrictions contribute to creating a “parallel” healthcare system for asylum seekers, influenced by various political, legal, and economic considerations. It also seeks to explore the implications of these systemic adaptations for health equity.

The structure of the article is as follows: We first present the legal situation and actual challenges in accessing healthcare among asylum seekers in Germany, followed by a theoretical background on system theory based on the German sociologist Niklas Luhmann. This theory is used as a conceptual lens to ground and contextualize empirical study findings from ethnographic work as we proceed in the article. After introducing the legal context and theoretical framework, we specify our aims and objectives, along with a detailed description of the methods employed and the sources of data collected. We then present the results of the ethnographic work and discuss the implications of observed individual and organizational behavior that foster systemic adaptations towards a “parallel” healthcare system.

### Lack of universal health coverage of newly arrived asylum seekers and segregation from the statutory system in Germany

Asylum seekers during the first 36^1^ months of their stay in Germany or until temporary or permanent residence permit is granted^2^ [12], rejected and subsequent asylum applicants and those who hold a tolerated stay permit^3^ have restricted access to health services under the Asylum Seekers Benefits Act (ASBA). The implementation of this law varies across federal states. In less than half of the territory electronic cards are issued to access limited services, while in the other half asylum seekers must request paper vouchers from the local government authorities to access this service spectrum [13]: §4 ASBA allows for immediate treatment of “acute” and “painful” conditions, vaccinations, care during pregnancy and childbirth, medically required preventive services, and care for unaccompanied minors [14]. Diagnostic or therapeutic measures, as well as medical equipment that exceed this framework (such as an MRI scan, antiretroviral drugs, a wheelchair, hernia surgery, chemotherapy, physical therapy, and psychotherapy) and are considered “indispensable in individual cases to ensure subsistence or health or are necessary to meet the special needs of children” (§6 ASBA) require an Application for the Coverage of Costs (ACC) to be issued by the provider and addressed to the respective government authority.^4^ During the initial reception phase, the regional council decides on each individual case, and in subsequent accommodation, the local social welfare offices of the city or district are responsible. §6 ASBA constitutes an option, not a legal claim and leaves room for interpretation [12]. Our focus in this article will be on this case-by-case decision making process under §6 of the ASBA, highlighting the discretionary powers exercised by various actors within the decentralized health and asylum system. Decisions often involve multiple care providers [15], as well as authorities at different administrative levels that act as purchasers and cost-bearers of services [16].

The medical and governmental prioritization processes lack transparency regarding the procedures and criteria for approving or rejecting benefits, particularly because there is no unified nationwide list of “essential” or “indispensable” services [17]. Decisions are therefore made without concrete guidelines and they take place against the backdrop of a discursive context that offers various frameworks of orientation, which can include contradictory demands. For example, from the standpoint of national politics, issues like state sovereignty and control over access to the national territory and wealth often seem to be in the foreground [18–22]. The ruling parties must balance their obligations under international conventions with budget management while also addressing societal demands for either greater inclusion or exclusion of non-citizens [23–29]. Medically, the right to health should be realized inclusively and progressively, without discrimination based on social, economic, or legal factors, including residence status ([17], cf. [30–32]). Decisions regarding the provision of medical services must be based on medical necessity. The measures should also – at least according to the guidelines of statutory health insurance – be efficient and effective. These few examples demonstrate that multiple, differing patterns of orientation may serve as contextual factors when deciding whether to allocate funds for asylum seekers.

The field of health care for asylum seekers is not only characterized by conflicting lines of discourse, their health care runs parallel or transversal to general healthcare; often formally organized in specialized facilities, predominantly located in remote reception centers at the outskirts of cities. Such walk-in-clinics were implemented as a reaction to unmet needs and various access barriers to the general system [15, 33] providing low-threshold access, e.g. by a centralized organization of the voucher system. These organizations were oftentimes built up ad hoc in times of perceived crisis during periods of large-scale immigration movements. Under the influence of various stakeholders, they were further developed mostly without central strategic governance from the general health system [15]. Although the organizations are highly heterogeneous in their material, organizational, and service delivery structures (see [34]), they all have developed along and hence operate in accordance with the restrictive legal requirements, as negotiated with their respective local government authorities. Therefore, refugee clinics can be seen as magnifying glasses, to examine the formal and informal organizational and other systemic consequences of legally restricted access to care. We will approach the question of organizational and systemic consequences of legal restrictions both empirically and theoretically.

### The system-theoretical lens as one analytical perspective

Given the aforementioned restrictive and exclusionary characteristics asylum seekers' healthcare in Germany has been described as a “parallel” healthcare system [15, 16]. Similar arguments, referring to stratified rights, structural violence and/or parallel care and financing structures have also been made for health care setups for asylum seekers in other countries, like Finland [35], Turkey [36, 37] or the UK [38]. We aim to further scrutinize and specify this thesis of a “parallel system” for the German case from a system theoretical standpoint, analyzing system level adaptions to restrictive regulations. Therefore, we have chosen to incorporate ideas from the German sociologist Niklas Luhmann to theoretically interrogate some of our empirical findings and determine whether they also indicate the existence of a “parallel system.” For this purpose, we will now briefly introduce Luhmann's understanding of systems, focusing on function, binary code, and polycontexturality.

Luhmann’s understanding of systems differs from the widespread understanding of socio-technical systems in the field of health policy and systems research [39], since in his theory systems are defined by the distinction they create – through their operations – between themselves and their environment [40]. Their structures are not permanent [ibid.]: Simplified you could say, they only “exist” when they are active. Therefore, from this perspective, systems are not organizations or made up of people, but rather what is done or communicated.

#### Basic assumptions: functional differentiation and binary code

According to Luhmann, society is made up of patterns of organized communication [41]. Around recurring problems and solutions, specialized communication spheres, so called functional systems (of for example law, science, economy or medicine^5^) have developed. Functional systems are not identical with organizations (cf. [42]): Economic operations do not only occur at the stock market or in a bank, but whenever something is paid for (cf. [43]). The medical system is not only operating when a doctor prescribes pain killers to an asylum seeker, but also when a social worker offers stabilization exercises for psychologically burdened asylum seekers, or when a security guard decides to call an ambulance when a resident reports chest pain.

Systems institutionalize different perspectives under which reality is dealt with [44]. Each system comes with an own world view and observes its environment differently [45], using programs and codes to simplify environmental complexity: a binary code determines how information is processed [43]. For example, legal communication, is oriented towards “legal/illegal” and science communication towards “true/false”. Every observation if filtered through this code and to one of its values further operations can connect [43, 46, 47]. (For an overview of the properties of systems that are of interest for this study, see Additional File 2). This connecting operations ensure the system persists.

A system operates on itself and generates its own structures in the process, which means the structures are not permanent but “at time only currently effective” [40] and they cannot be imported. Not everything from as system's environment is meaningful to it. But it must be flexible, as it is often structurally coupled and therefore interdependent with other systems [40]. In cases of such coupling, something from the “environment”, that the system would typically respond to indifferently is transformed into information that it can process. It might adapt or modify its structures accordingly. In our case, the medical system might initially be indifferent towards a change in political “climate” regarding specific patients, but – because of its coupling with the legal and economic system – it “understands” a lack of re-imbursement for planned or provided health services, if certain procedures are not followed, and might transform accordingly.

#### Properties of the medical system

Luhmann discussed the medical system only in a few short articles [48], describing its function – with a service delivery focus – as “restauration of damaged health” [49], leaving out health maintenance and promotion which are part of the WHO´s health system definition [50, 51]. According to Luhmann’s system theory, the medical system operates along the binary distinction (or code): “ill/healthy”. It reacts to – via its programs – detected “illness” (or potential illness). When “health” is detected, no action is required, so no further operations follow [43]. Important to us will be: This "coding is not just any structure, but the guiding difference of the system, which all operations follow" [ibid.]. Changes in this respect lead to a transformation of the system [ibid.]. If and how such adaptions happen, is decided within the system, since “other systems cannot make programs for diagnoses and treatments. The political system may of course try to influence the medical system, but it is the medical system that decides how it will react to such attempts” [45].

#### Polycontexturality

Conventionally, we associate most organizations with a specific functional system [52] like hospitals and medicine, assuming that the respective rationality takes precedence there. However, every hospital is also subject to financial, legal or political observations and decisions, and may also consider educational or scientific issues (cf. [45]). The systems “observe each other, […] relate to each other but also distance themselves” from one another [53]. In modern societies, communication increasingly needs to refer to different systems and their respective codes and evaluative principles simultaneously, which is called “polycontexturality” [53]. Like we mentioned, Luhmann’s approach helps to distinguish perspectives that are in reality often intertwined [42].

Theoretically, the contextures in such settings are ordered “heterarchically”, no perspective is per se seen as more important. That does not imply they are equally important or influential, “but differences in degrees of domination” require explanation (cf. [42, 45]). Conflict and tensions may occur, especially when systems with incompatible rationales observe their interdependency [52]. “A decision that is medically correct can be problematic from a financial or legal point of view. A legally and financially correct decision may not be compatible with the rules of the medical profession.” [45]. The question is how the contextures are being brought into a workable arrangement.

#### Explanations regarding the use of this theoretical approach

In this article, we adopt a predominantly inductive, qualitative, and descriptive ethnographic approach to provide insights into the public health issue of translating specific access restrictions to care into medical practice. In addition to this methodological approach, we use the described theoretical concepts to deepen our understanding of particular empirical phenomena. With the help of system theory, we want to 1.) categorize prioritization-criteria regarding health services for asylum seekers and 2.) better understand the significance of adaptions to restrictive regulations for the medical system.

We have chosen Luhmann's rather rigid, autopoietic system concept because it facilitates the assignment of specific rationales (logics, moral codes) to distinct systems. This allows us to determine whether a given decision criterion for medical services is primarily legal, medical, political, or economic in nature (in chapter Priority-setting as polycontextural assemblage). Luhmann's theory helps to first clearly differentiate the rationales or perspectives of various systems. Then we can analyze how these systems interact, connect, depend on each other, and transform. As stated above, systems are not seen as bound to organizations and their professionals, they are not something fixed, but active when their rationales are applied, regardless of who applies them. The political system is active when power is exercised and binding decisions are made – in our case, when enforcing the migration-related political rationales of ruling parties. The medical system is active when illness is detected and actions are taken to restore damaged health. This means that, in a strictly theoretical sense, when actions are taken or decisions are made, that do not serve this purpose, it is not the medical system that is active. For example, if a *physician* considers a patient's prospects of staying in the country and thus contributing to the host society later, when deciding whether to ask for cost coverage of a therapeutic measure [54], the *political* system would be considered active in that instance. Therefore, this theoretical approach also allows us to understand when actors switch between codes or rationales, activating different systems logics.

Additionally, Luhmann’s systems theory aids in assessing whether the gathered empirical evidence supports the thesis that a “parallel system” of medicine for asylum seekers has emerged in Germany (see chapters Bureaucratization and micro-political struggles over cost coverage: Reflections on a de-politicizing effect of organized forms of health care for asylum seekers and Reflections on theoretical aspects of a medical sub-system for asylum seekers' healthcare). To better delineate the two analytical approaches (qualitative-inductive/meta-theoretical reflection), we will use the term “medical system” when we refer to Luhmann’s sociological system theory, and for more general reflections we will continue to use the term “health system”.

## Aims and analysis steps

We want to explore processes of health care provision for asylum seekers in the face of legal restrictions, focusing on the individual, organizational and systemic translation process of those restrictions into practice. Our leading questions are:

1. How does the prioritization process for medical services – according to §6 ASBA – work?
2. What rationales are being invoked while enacting the legal restriction in daily practice and how do the actors bring them into workable arrangements?
3. What are the systemic consequences of the translation of legal restrictions into practice?

After presenting our methods, the analysis will be structured as follows: We will firstly map the organizational “architecture” of the decision-making process for granting and denying medical services, shedding light on the nature of structural adaptations within organizations. We will identify the actors involved in service delivery decisions and explore how professional tasks, responsibilities, and power relations within and between government and healthcare organizations are affected by the restrictive legal requirements. Secondly, we explore prioritization criteria of the decision-makers to assess which perspectives are being actualized in the prioritization process. Thirdly, we will theoretically discuss some aspects of the empirical evidence pointing to changes within the part of the health system that is specialized to care for asylum seekers. Finally, we will ask for the implications of these findings for health equity efforts.

## Data and methods

### Field sites and recruitment

We chose an ethnographic approach that included non-participant and participant observation, in-depth interviews, and document analysis. The study was deliberately conducted at two very different outpatient-clinics, within shared accommodation centers, providing care mainly for newly arrived asylum seekers in their first 18 months^6^ in Germany. The centers were in two federal states, differing by size, governance and health financing responsibilities, organizational structure, medical disciplines within the outpatient clinics, and their self-image (Table 1).

**Table 1:** Characteristics of study sites

	Site 1	Site 2
**Facility type**	First reception centre	Shared accommodation centre
**Number of residents**	1760	380
**Governance**	State level	Municipal level
**Health financing**	State-level authority	District welfare agency
**Medical disciplines regularly represented in the outpatient clinic**	General Medicine, Gynaecology and Obstetrics, Infectious Diseases, Psychology, Paediatrics	General Medicine, Gynaecology, Paediatrics
**Number of medical professionals simultaneously present per day**	8	1–2
**Number of GPs simultaneously present per day**	2	1
**Number of patients seen by the GPs per day**	up to 30	up to 18
**Number of nurses simultaneously present per day**	3–5	1–2
**Nurses participation in consultations**	Mainly administrative responsibilities	Own consultation hours of nurses, and assistance in all medical consultations
**Self-description**	Providing immediate, basic care	Providing closely-knit medical support with a human-rights-based approach

In these different settings, characterized by both personal and organizational diversity, the same national legislation is being implemented. We not only examine the different ways in which the law is enacted at those sites, but also aim to identify communalities and overlaps in structures, processes, and their effects. This analysis will help to design an ideal typical model for the cost coverage process for health services for asylum seekers.

### Recruitment, ethics approval and consent to participate

We selected two maximally different cases from the existing networks from previous studies of the second author. We reached out to the managers, scheduled personal meetings to inform about the ethnographic method and objective and obtained their cooperation and consent. The nursing teams were consulted and agreed to allow the first author, a social and cultural anthropologist, to participate in their daily routines. As physicians rotated, each was informed about the study and confidentiality prior to their shifts and verbally consented to the researcher’s presence. Patients were also informed and explicitly agreed to have the anthropologist present during their consultation. Where consent was not given, the anthropologist would leave the room. Interview participants were provided with written information and signed consent forms. The study was approved by the Ethics Committee of the Medical Faculty of Heidelberg University (S-287/2017). To prevent recognition or stereotyping, names of individuals, organizations, and nationalities will be changed in this text.

### Field exploration and documentation of Applications for Cost Coverage (ACCs)

The first author spent a total of three months in the field [55] between August 2018 and September 2019. She worked in one facility in two blocks and in the other regularly on specific weekdays over an extended period. She participated full-time during opening hours of the clinics. Relevant documents were sighted after opening hours. Observations, conversations and informal interviews [56] were captured in a handwritten field diary. Written material concerning entitlements and ACCs was collected (e.g., vouchers, outgoing ACCs, entries in patient records, answer letters from authorities). At both sites 110 requested services were documented during the field phases.

### In-depth-interviews

Prior to and during the field phase, we employed a purposive sampling technique to recruit medical and government professionals – who were regularly involved in the application process – for in-depth interviews. Semi-structured, problem-centered interview guidelines were developed iteratively, taking into account fieldwork and initial interviews. This allowed us to gather expert knowledge and experiences related to the ACC process from ten health professionals (Nurse/Doc) and five government administrators (Admin) (see Additional File 2). Additionally, we conducted interviews with six translators to provide contextualization, as they are directly observing the cost coverage processes and negotiations within the clinics on a daily basis.

### Analysis of interviews and field protocols

Field notes and protocols were digitized, resulting in a total of 112 pages. Additionally, 76 h of interview audio recordings were transcribed using f4. The entire dataset was then analyzed using MAXQDA. We chose an iterative, mixed deductive-inductive approach (cf. [57]). Drawing on techniques of a structuring qualitative content analysis according to Kuckartz [58], we initially derived overarching categories from the questions and data. Subsequently, the material was further categorized into sub-themes emerging from the data. During the analysis, we also paid attention to word semantics [59] regarding the legal frame. To reconstruct the ideal typical prioritization process of medical services, the relevant information was extracted from the data using strategies for analyzing expert interviews [60]. Furthermore, statements concerning prioritization criteria of health professionals and government administrators were screened for assignability to a functional system and summarized (see Table 3).

## Results

### The ACC process: actors, steps and responsibilities

The legal restriction on health services has to be “translated”, i.e. enacted and made understood in the health system [61]. This translation involves the introduction of uncommon structures controlling access to care, specifically the involvement of government actors in healthcare financing and the implementation of a multi-step prioritization process. These changes require new forms of communication and decision-making, resulting in transformations in routines and responsibilities within both healthcare and governmental administrative organizations.

**Fig. 1:**
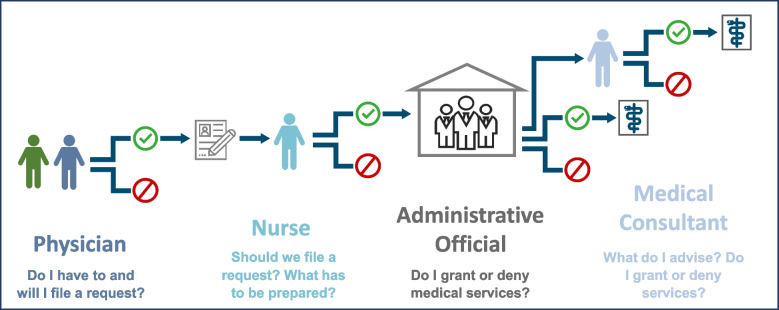
ACC process flowchart

Figure 1 illustrates a typical application process for cost coverage for specialist visits, specific diagnostics, medications, and therapies (under §6 ASBA) for asylum seekers. In the subsequent text, we will delve into the tasks, responsibilities, and decision-making powers of these actors, supported by quotes from our qualitative data.

#### Physicians

##### Do I have to file an Application for Cost Coverage (ACC)?

In each consultation with an asylum seeking patient, physicians must decide whether they are dealing with an “acute” case (see also remarks in chapter Translation of the law: notes on semantics) and can proceed as medically indicated right away or *have to file an ACC* first. We have noticed various factors that are taken into consideration in deciding whether an application is necessary, for example the personal interpretation of the law plays a role or considerations of the costliness of a measure (see Table 2).

**Table 2:** Factors influencing ACC submission decisions

Factors for determining the necessity of an ACC	Notes and Quotes
**Knowledge of the legislation**	Of seven care providers explicitly asked about legal knowledge, three were familiar with the legal text, one could cite it correctly. However, all providers were aware that restrictions apply.
**Interpretation of legal terminology**	The interpretation of the legal term “acute” could be challenging: “*With so many things, the line of acute is fluid. With an acute infection it is clear, but if you have back pain it is fluid, do you need it or not?” (Doc2P)*
**Narrow or broad interpretation of the law**	What was considered an “acute” condition differed between physicians. “The” law could at times be intentionally interpreted more strictly or flexibly.
**Disease or intervention specific criteria**	Based on previous experiences, caregivers considered whether applications had been necessary for certain services. *“We have to file for orthopedic diagnostics for back-, joint-, feet pain” (A3E); “anti-retro-viral-drugs and chemotherapy” (A2E); “diapers for adults” (LK01P)*
**Knowledge of the practices of local authorities**	Some authorities require an application for every drug, others only for particularly expensive diagnostic and treatment procedures (see also Wahedi et al. 2020).
**Costliness of the measure**	Health professionals assumed that a cost coverage request would likely be necessary for high-priced medications and therapies.

##### Will I submit an application for cost coverage?

If filing an ACC has been deemed necessary, physicians decide if they *actually file*. We observed a continuum of reflections and practices ranging from anticipatory obedience to advocacy for patients. An orientation on imagined prioritization criteria of authorities, could lead to hesitant or reluctant ACC practices. As in the case of a 28-year-old patient from West-Doangia, with a movement restriction of the left elbow after trauma, who showed a physiotherapy prescription from the orthopedic clinic to the general practitioner of the outpatient-clinic, who – obviously deciding not to file a request for him – said to him:

*They prescribed physiotherapy, but is not paid, so you have to do it on your own, do some weightlifting with low weights or body weight! (FNE_0719)*

In facility 1 health professionals aiming to increase the own “success rate” of ACCs had implemented uncommon, additional prioritization loops: Two doctors weekly checked all referrals of their outpatient-clinic colleagues as well as letters from specialists presented by the patients (e.g., recommending an operation) and decided whether those ACCs would be forwarded to the government administration at all (A2E, LKE).

In weighing up arguments, many health professionals pointed to the fact that submission decisions are made against self-defined limits of medical necessity:

*[…] everyone has a different attitude towards it. But I think you have to consider what the patient really needs or not. Because if we start to request all kinds of things for everyone, then we simply become less credible, which then also harms those who really need something […]. (Doc1E)*

*[The oncological condition is] too bad, it's too expensive, promises little success. Of course, the patient says: I want everything, but first the [medical] specialists check whether it is necessary and promising. (FNNurse1P_0818)*

Some health professionals explicitly distanced themselves from non-medical rationales by means of their professional identity, they believed it was their duty to advocate for their patients in filing requests:

*My personal opinion is: It is simply our duty to represent the interests of the patient and we must persistently file requests for what we believe is medically indicated. And then another level must decide. (Doc1E)*

In the ACC letter, physicians are required to provide a justification for the necessity of the measure. Medical documents, such as letters from other physicians and diagnostic reports, serve as evidence. We identified communicative challenges arising from differing communication styles and technical language used by health professionals and administrators. To streamline ACCs for referrals to specialists, at both sites simplified procedures had been developed, including the use of standardized forms that did not necessitate a free-text letter.

#### Nurses/medical assistants

*Should we file an application for cost coverage?*

Nurses´ involvement in the application process differed between field sites, but they frequently assumed an advisory role, especially regarding the question if an ACC should be filed. Their assessment was guided by three main factors: a) perceptions of their mission; b) assessments of patient need; c) collective and personal experiences with previous ACCs, even if those were inconsistent.

Their approach to filing can also be seen as a spectrum, ranging from efforts to increase the success rate of filed ACCs to advocating for patients and their needs. Here we also see anticipatory obedience, when nurses try to avoid or speak out against submitting applications with perceived low chances of success:

*If we would file all the requests that we, our doctors, consider necessary […] then there would probably be one folder with approvals and twenty with rejections. (NurseIE)*

Health professionals reported time-consuming, sometimes exhausting bureaucratic processes and negotiations with authorities. Despite their efforts, the outcomes could still be frustrating. Our observation suggests that individuals tried to preempt this frustration by anticipating the likely outcome, thereby avoiding investing energy in endeavors they believe had a low chance of success based on past experiences. In the following quote, a colleague reflects that this approach, coupled with a sense of fatigue, has at times resulted in a lack of further efforts being made:

*[…] we totally limited ourselves because we always thought it was pointless anyway, and because it was super exhausting and then it was always rejected [...] we did far too little, I think […] because it's exhausting and because you often have to fight against so many windmills anyway […] and then you didn't waste your energy on a three-page, um, psychotherapy, um, application that was then rejected, you could put your energy into something else. (Nurse1P)*

Just as among the doctors, there were also statements from nurses who argued that ACCs should always be filed, regardless of their chances of success, if only to demonstrate the need:

*I think in any case we should try. If only to simply announce the need, to show what people have. Even if we expect it to be rejected – even more so! To simply show: Hello?! But it is necessary! (Nurse2P)*

*What has to be prepared or followed-up upon?*

Nurses hold the administrative and communicative responsibility for ACCs. They compile documents and forward them to the locally responsible officials. They follow up on the ACC with varying frequency and inform physicians and patients about the outcome. Only special and conflicting cases were followed up by physicians. Nurses’ involvement in formulating ACCs differed between the facilities, from close involvement, even pre-formulation of ACCs (facility 2) to only handling additional forms (summary form, release from confidentiality) (facility 1).

#### Administrative officials

*Do I grant or deny the requested medical service?*

In order to implement legal restrictions, government administrations have taken on new responsibilities, leading to the creation of new positions dedicated to processing requests in accordance with §6 ASBA. At our sites, individuals with backgrounds in social welfare organizations were predominantly recruited for those positions. The role of these administrative officials involves reviewing ACC material and occasionally requesting further information or justification from healthcare professionals. Their decision-making authority varies, as they may either a) independently make decisions on all or some measures, b) consult external medical experts for guidance, or c) delegate decision-making to external medical consultants.

At field site 1, the administrative personnel autonomously made decisions on referrals and ACCs. During the study period, external medical advisors were only consulted for complex or expensive cases, or when patients had already filed an official objection against a previous decision. In contrast, at site 2, administrators had limited decision-making autonomy. The majority of decisions were delegated to medical consultants from the public health department. Administrators were only responsible for decisions on referrals and measures valued below €250; their main role involved organizational tasks such as communication with consultants, patients and medical facilities.

Decisions of administrators are made on a case-by-case basis: *“[…] you simply say that's what we do now, but that doesn't mean that we do it like that in all cases” (AdminE2).* In the absence of medical training and binding guidelines, administrators must also interpret the law, frequently employing an assemblage of medical and non-medical prioritization criteria, that often showed an interdependency with asylum logistics. For example, when considering non-medical aspects like the ability to travel or the stage of the asylum procedures, which was – according to an administrative official – what made *“acute and necessary in the Asylum Seekers Act a completely different term than 'yes, he'll die otherwise' in the medical profession” (AdminE2)*. She described a case, where this interdependency between medical decision making and asylum logistical considerations becomes apparent:

*I had a patient who had to have her leg amputated because she had bone cancer, and we were told [by a physician] that further treatment would have to follow. Immediately! [Our] medical consultant said: we can wait six weeks and in the six weeks her status shifted, she was obliged to leave the country [so I rejected, but then] due to a combination of illnesses, her ability to travel changed. […] then I had to approve. [...] and when she was fit to travel again, then the question was again, where do we stand now? (AdminE2)*

Generally, we observed, that the relationships of administrative officials and health professionals differed at our field sites in terms of personal acquaintance, contact density and working arrangements.

#### Medical consultants or assessors

##### Do I recommend the approval or denial of coverage for medical services?

In some prioritization decisions medical consultants are involved, they can be of any medical discipline and have different affiliations and decision-making powers.^7^ At our field site 1, they were affiliated with a health insurance-related organization, providing medical advice to administrators on a fee basis when requested by the administrators.

*I said that we need experts, we simply need medical expertise that tells us, as non-medics, whether it is the next step in treatment or not, whether it is necessary, whether it can be postponed, whether it is palliative, whether it can be done in the home country or not? […]. We still have to decide whether we finally grant the benefit because of it, they´re still our discretionary decisions […]. (Admin2E)*

At site 2, consultants affiliated with the public health authority were responsible for making binding decisions on ACCs. Only a small number of patients were called in by the medical consultants, so the assessment at both sites was predominantly based on written material without patient contact, which was a fact that many health professionals criticized. While health professionals had written and sporadic telephone contact with administrators, they could not directly contact the consultants. Consequently, the consultants' prioritization process remained opaque to professionals and administrators. Detailed explanations were rarely provided; with standardized text blocks being sent to administrators for relay to health professionals. These texts commonly referred to factors such as “acuity”, “pain”, “postponability”, or “danger to life”. This complicated the task for administrative staff to explain to healthcare personnel why a requested medical measure was rejected.

*[...] the letter is almost always the same, it says [...]. At the present time, knee surgery is absolutely necessary and cannot be postponed” or “At the present time, knee surgery is not necessary and can be postponed, it is not a pain therapy” [...] some take their time and write it out a bit, but it's never more than one page [...] and then it's extremely difficult for me: Well, what do I write in the rejection? (Admin1P)*

Upon closer examination of the overall process, it becomes evident that at each of the three to four steps (see Fig. 1) of this prioritization process, a decision to withhold services may potentially be made. From the healthcare side alone, from one up to five professionals can be involved in filing decisions.^8^ Ethnographic exploration has revealed instances where ACCs were not filed at all, although the exact number is difficult to assess. Some health professionals may document their decision not to file within the patient documentation system on the same day, but not all do so, and there is no comprehensive documentation of these internal decisions. At the government level, an additional one to three actors are involved in deciding whether a requested service – deemed necessary by a physician – is granted. Before this study both outpatient-clinics had no patient-wide documentation on how requests were decided by the government.^9^ In our quantitative analysis of 110 requested services during field time in both facilities, 51 were granted while 30 were denied (see Additional File 4). The standard duration of the ACC procedure varied significantly, with informants reporting processing times ranging from five days to three months. During our documented times, processing times ranged from one to 151 days, with a median of 12 days.

Processing ACCs poses a significant administrative challenge for healthcare and government organizations. Staff members gradually acquire the necessary knowledge of the process and continuously negotiate its various aspects. Our informants report that outside their facilities, many health professionals have not acquired the necessary procedural knowledge and experience to (successfully) file an ACC. This creates a barrier for asylum seekers in accessing the general healthcare system.

### Translation of the law: notes on semantics

The legal terms “acute”, “painful” and “indispensable” for health are processed by the medical and administrative system. Since the term “indispensable” is rather vague, both systems seem to refer mainly to “pain” and “acuity”, which are – albeit legally disputed [62] – familiar in medicine. However, in regular business these distinctions are not clearly conceptualized to decide on activity or inactivity of the system. We observed multiple translations at the administrative and medical level, like the opposite of “acute” being “chronic” or “pre-existing-condition”, as synonyms for “acuity” terms like “emergency” or “life-threatening” were in use (see forthcoming article). Both involved parties were aware the other party might define differently and considered that in their communications (and decisions).

We have not only observed the described stretching of the concept of acuity, but also an expansion of its application. Legally, the determination of whether something is acute or not (§4 ASBA) is intended to decide if an ACC must be submitted before initiating healthcare measures. However, we have noticed something peculiar: The notion of "acuteness" is also regularly taken into consideration in §6 decisions – the paragraph that explicitly applies to cases where conditions are *not* deemed acute or painful and therefore require an ACC. In these cases, too, health professionals ask themselves if it is “acute” enough that the ACC might be successful, and administrators ask if it is “acute enough” to be granted (see also Table 3 below).

### Transformation of power relations and hierarchical structures

The process of prioritization, which involves previously external actors in health financing, represents a structural change that leads to shifts in power relations. Specifically, the position of medical doctors seems to be weakened, since professional autonomy is unusually restricted.

*[...] that it has to be approved again by the welfare agency or by someone else, […] is of course very strange for me, because if you come to my practice […] I just write you a prescription […] and don't think about it any further. (Doc1P)*

Physicians are usually guided by knowledge, technical standards and their professional ethics (cf. [63]). Asylum seekers' health care is additionally oriented towards – via law and administration – already specifically reduced complexity (cf. [64]). One of the nurses reflected her feelings regarding those restrictions, saying she could not treat everyone the way she would like to – as a professional and as a human being:

*[...] the rules are simply different [than] for me as a German [..] in the German health system. [...] so you can't just do what you would like to do, or what is logical. […] what is logical as a fellow human being or so, or also as a medical or staff person […] but it is always a little cracked, […] a little cut off, always a little limited. You always have to improvise [...] you always have to ask somehow whether you can get it or not and that in general […] the Asylum Seekers Benefits Act medicine, somehow only does emergency things in the first reception [...]. (Nurse1E)*

Administrative personnel also perceived this new power structure, which places them in the position of making decisions on medical measures, as unconventional.

*[...] I'll put it very simply: a university professor doesn't like to be told by a typist that he's not allowed to do that. (Admin2E)*

In some cases, power struggles were played out openly, e.g., when physicians had prescribed anti-retro-viral drugs, for which they refused to file requests, because they regarded HIV medication as self-evidently necessary (Nurse1E) or when health professionals helped patients to officially object rejections of ACCs. Reversely it has been reported that the administrators resorted to using or threatening non-payment or recourse as a strong argument to enforce compliance with their rules (Doc3E).

In principle, health professionals seemed to consider the decisions of government agencies to be legitimate and binding. Nevertheless, even after integration of these agencies into the healthcare system, they continued to be viewed and portrayed as external entities, especially when rejections needed to be communicated to patients:

*The patient says: “[…] but I'm sick and I'm in pain" […] then I always have to say "Yes, but the administrative council doesn't pay for that, that's not (…) we would pay for that* >*laughs*< *(Nurse2E)*

Power shifts occur both between and within the involved organizations. Where process innovations were inevitable, it was the individuals involved in the daily reimbursement processes who played a key role in shaping them. These individuals were not necessarily physicians or senior administrative officials, but rather nurses and administrative staff. They were instrumental in designing communication chains and formats, thus exerting significant influence on power distribution mechanisms.

*So, we had to somehow find our way around and there was no one who stood there and said: you know, this is how we do it now or this is the patent, but we had to muddle through ourselves. (Admin2E)*

*[We had to deal with questions] that were never asked before by anyone in the administrative council, i.e., reimbursement of costs, um, refusal of benefits, expert opinions, […] framework agreements. […] then I sat down with [my superior] and tried to find solutions. (Admin2E)*

In healthcare organizations we have observed a hierarchical shift towards nurses. Although physicians are principally responsible for prioritizing needs, making filing decisions and writing applications, nurses are considered as internal experts due to their more consistent presence and/or communicative responsibilities for ACCs towards government, physicians and patients. This gives them interpretative power, as their understanding of the law might be accepted as its content. A physician explained his unfamiliarity with the legal framework in the following way:

*[…] I rely on the (..) on the staff who are there, yes, that I say, for example, “Are we allowed to do that now?” or someone (..) is perhaps positive for hepatitis C or positive for HIV: “Are we allowed to give him super-expensive treatment or not?” and they then tell me: “No, only if he is ill, if he (...) yes, if certain things are present.” They are the ones who are in contact [with the government]. […] their experience is more important to me than if I look it up on the internet because they can tell me exactly “Yes, it's not possible”. (Doc1P)*

Nurses could exert considerable influence on filing decisions by way of advice, follow-up and general personal engagement. They could advice doctors according to their experiences or withhold such advice:

*Sometimes I say to the doctor who writes that it must be done, that the [specialist's] letter only recommends it and that it has no chance anyway, [then he writes something else]. (Nurse2E)*

*[…] when Dr. Böhnlein, for example, wants to request something, because he likes filing [ACCs], I know in advance that it won't work [...] But I don't tell him that. [...]. If he asked me, […] I would tell him […]. (Nurse2E)*

Nurses could closely follow up on ACC cases after filing or refrain from doing so and they could influence processes by in- or decreased engagement for a repeated request or objection.

*[...] sometimes I don't see why it's not approved and then* >*quieter*< *I ask again. [...] if I just don't see that (...) why it's not approved, because I think it's dangerous or because I think it has to be done (Nurse2E).*

It was even reported, that nurses had withheld ACCs in individual cases, if they assessed a high probability of them being rejected.

*[...] I know that we have sometimes not done [ACCs]. Even if the doctor said so, we decided together – it's not worth the trouble. (Nurse1P)*

Power dynamics can also shift among physicians within an organization, based on their social connections with the nurses or increased involvement in prioritization processes and related negotiations with officials. We encountered physicians and nurses who had enhanced their credibility with government staff through extensive networking, thereby bolstering their position when making requests (FNDoc2E_0819).

### Priority-setting as polycontextural assemblage

Service delivery decisions for asylum seekers are regularly not solely based on medical criteria, but rather a combination of different rationales. In each case, at every step health professionals and administrative actors negotiate within themselves and with others which of them will be given precedence.

*Doc3E: [...] if it can wait until the transfer, I write until the transfer, but if he came maybe three or four times and he's suffering, […] he's not well, he can't cope with everyday life […] then we do these [ACCs].*

*Admin2P: [I reject] everything that is not acute and can be postponed, so someone who comes with an eye disease that he has had for two years, I must reject, because he comes from the Mandi States, to put it bluntly. Or we have people [who already have a deportation appointment to another European state according to the Dublin agreement] but they stay here because they need a hip operation, then I say, he's been walking around with this hip for five years. Of course, it's not a good condition in the long run and it will probably get worse, but according to the law I'm not allowed to approve. […] It always has to be assessed, what prospect of staying the person has and can I therefore – as a prognosis into the future – grant the service. That is the difficult position, we are in.*

To gain an overview of the multitude of contextures that are actualized in prioritizations of health care services for asylum seekers, we consolidated our findings from observations and interviews, aiming to differentiate between perspectives (see Table 3). We assigned observed prioritization considerations or criteria to four functional systems: legal, medical, economic, political, and the political subdivisions of administratively relevant logics as well as migration-political considerations. Additional criteria that could not be assigned, such as patient-specific ones on medical side and formal ones on administrative side as well as the possible influence of social contact and connections with patients and among decision-makers are omitted here.

**Table 3:** Polycontextural assemblage of prioritization criteria

	Filing decision of medical organizations	Cost coverage decision of government administrations
**Legal System** (legal/illegal)	• Emergency? (definition/s) • Acuity? (definition/s) • Pain? • Necessity (definition/s)	• Acuity (definition/s) • Pain • Pregnancy • Age (child?) • Necessity (and its definition/s) • Orientation on court decisions regarding withheld services
**Medical System** (ill/healthy)	Nature and degree of medical necessity • Perceived level of suffering • Considerations regarding possible postponement of treatment • Consequences of denied treatment • Effects of treatment: curability, profitability for patients	• Distinction between acuity and chronicity • Distinction between pre-existing condition and those acquired domestically • Urgency of treatment (postponable?) • Nature of treatment (e.g., frequency, duration?) • Medical prognosis/prospect of healing • Consequences of denied treatment • Duration of treatment (completed before transfer or deportation?) • Possibility of treatment in country of origin or deportation
**Economic System** (payment/non-payment)	No explicit de-prioritization, but reflection on • Costs of services • Solidarity-principle of health insurance, protection of tax-money • International responsibility (e.g., making amends for Western wrong-doing, inequality that we can or cannot compensate) • Concerns about becoming liable to recourse in case of a missed but necessary ACC	• Closer examination regarding expensive services (starting at approx. 150–200 €) • Always cheapest drugs, smallest amount • In the case of pending change of jurisdiction (transfer to another state), tendency to delay (if medically possible) so that the next instance may cover the costs • Reference to protection of tax-money • Concerns about becoming liable if own decisions are regarded as too generous in the face of legal restrictions
**Political system** (powerful/powerless) represented by administration = responsible for binding decision-making	• Prior experience of approval or rejection	• Responsibility to implement the law • Consideration of possible effects of own decisions on asylum process
Administrative rationalities	• Organisational effort (weighed against chances of success) • Compliance with extended restrictions for subsequent applicants and patients to be transferred or deported in a timely manner	Asylum logistics and process stage • Registered asylum seeker? (Without asylum application, access to services is usually limited to acute care) • Pending relocation (transfer)? • Subsequent application? • Pending obligation to leave the country? • Keeping or restoring fitness to travel Administrative procedures • Timely and comprehensive ACC • Filed objection – new audit, possibly changed outcome
Migration-political considerations	Heterogeneous attitudes towards political agendas • Concurrent rejection of discrimination according to countries of origin • Observable categorisation of patients according to migration political categories: e.g. (flight) motives or prospect of staying (no verifiable de-prioritization, further studies needed)	• Prognosis on prospect of staying depending on country of origin (safe countries?) • Flight motive classification (especially negatively valued: imagined health seeking migration)

Among health workers, the most frequently mentioned criterion for deprioritization was the negative assessment of an ACCs chance of success (as mentioned earlier). These assessments are determined by communicated or assumed criteria of administrators. Health workers could name these presumed criteria (see forthcoming article). Even if the decisions remain incomprehensible from a medical point of view, an explanation from an administrative point of view is derived, meaning is generated, and operations can connect. Consequently, all criteria used by the administrators may potentially influence the decisions made by health professionals.

According to Luhmann, criteria from one reference system cannot be applied in another. This means that if the observational perspective changes, so does the observer. Systems exist when the relevant rationalities are operationally applied. In healthcare for asylum seekers, several observational perspectives coexist, observing themselves and others. The protagonists might be particularly aware of the multiplicity of perspectives (polycontexturality), since in the ACC process the pressure to justify one’s choices or to assimilate and adapt to other rationales seems omnipresent.

Fights for primacy of the perspective that is considered one’s “own”, be it medical, legal, political, or administrative, are a daily occurrence in asylum seekers' health care. A perspective that is considered as external can be accepted, leading to the incorporation of justifications from this “other” side. Alternatively, other perspectives can be rejected, because they conflict with one’s professional mission and the function of one’s primary reference system: Government employees feel responsible for aligning healthcare with economic and migration-political rationales, while health professionals should provide non-discriminatory care based on medical grounds. The following two quotes vividly illustrate the struggle to stay true to one’s “own” mission. The actors recognize the distinct rationales they encounter within their field and often feel compelled to assert strongly the systemic position they believe should be their primary one. While rejecting conflicting rationales, they must still engage with them to establish boundaries. It becomes evident that previously “foreign” rationales, even when dismissed, remain cognitively present in their daily work, and one can imagine the pressure they exert:

*Admin2E: Doctors often say, “it's necessary, it's necessary” because in the chain of indications, […] of treatments that an insured patient undergoes, it's always like that […] that's not the case here and that's why this term “yes, of course it's necessary, otherwise he'll die”, we all die at some point. […] it's not my fault that he dies because I refuse the service, it's his illness and um – this acute and not postponable – that's a term that refers to the fact that if it can be postponed for two months and the patient is obliged to leave the country in four weeks, then he has to leave and can then organize further treatment in his home country […]*

*Doc1E: It was suggested [by government officials] [to] consider whether the person has any prospect of staying at all. Because those without […] are granted very little. To reduce effort, [they appealed]: “[…] if this is a patient from the Mandi states, who obviously comes here for medical reasons. We'll deport him anyways. You could save yourself the trouble.” And frankly, I think that's […] a bloody mess. […] To make such a request to the doctors. They are simply trying to shift the solution from a political problem to the medical level, [which is not possible]. Of course, they are trying to take away the incentive to come to Germany for health reasons and to apply for asylum here. And I can also partly understand that our system could not afford to operate on everyone who does not have the resources in their home country. But then they should find a political solution.*

## Discussion

### Reflections on distinctive healthcare for asylum seekers in Germany

The legal barrier to health care access for asylum seekers has repeatedly been described and criticized [14, 65–68]. Our empirical observation shed light on complex structural, organizational and micro-political consequences of this barrier. In translating the legal restriction into practice, structures, and power-relations in this part of the health system are transformed. The involvement of actors outside of the conventional realms of the health system into decision-making on approval of health services and coverage of related costs leads to the implementation of a cross-sectoral prioritization process, which presents all involved actors with novel tasks and responsibilities while also reshaping power dynamics both between and within the organizations involved. Physicians perceive a decrease in medical autonomy, as they are required to seek permission for many actions. Through networking with colleagues or administrators, some physicians may be more successful in asserting their claims than others. However, in the daily operations, nurses often hold expert status regarding ACCs and can exert considerable influence on filing decisions and follow up. As process development and renegotiation take place at the local level, involved health professionals and government administrators can influence power distribution mechanisms. Due to the lack of transparent and comprehensive guidelines and wide discretion, *all* involved actors at *all* levels wield translatory power in interpreting *the* law (cf. [69]). Many individual discretionary decisions made by a greater number of actors than usual stand between an asylum-seeking patient and healthcare services.

Looking at the prioritization criteria, it is evident that actors weigh multiple rationales in their decision-making process. In healthcare decision-making, it is common that multiple, sometimes ambivalent or contradictory rationales intersect (cf. [70]). Managerial or economic considerations may need to be balanced with medical factors [71, 72]; or legal and medical aspects must be navigated, for example in end of live care [73]. What distinguishes asylum seekers' healthcare is that parallel funding, and – in case of outpatient clinics – governance structures, link it more closely to government authority than is usually the case. This results in different incentives and disincentives for care provision as well as additional rules and practices that must be taken into account. Many of the multiple applied prioritization criteria used in financing health services for newly arrived, rejected and tolerated asylum seekers differ from or are in direct conflict with those of the statutory health insurance system for the general population. Legal care restrictions allow for the implementation of explicit and implicit control mechanisms that align service provision with political rationales, such as cost-saving, deterrence of asylum seekers, creating disincentives for refugees from certain countries to immigrate or stay, or encouraging “voluntary” returns ([22, 74], cf. [75]).

Furthermore, institutions within the general healthcare system, such as hospitals and resident specialists, are generally unprepared and often overwhelmed by the administrative hurdles of providing care for asylum seekers, which increases access barriers. However, despite these challenges, asylum seekers are still offered essential and frequently more than restricted care. Within the margins of discretion of all its enactors and dependent on their engagement, care similar to that of the majority population *is* possible, but: “They don´t get everything, they only get everything with immense effort” (FNNurseP_0718). The organizational effort not only leads to delays in treatment but may also have a depoliticizing effect.

### Bureaucratization and micro-political struggles over cost coverage: reflections on a de-politicizing effect of organized forms of health care for asylum seekers

Paradoxically, in the organized form of asylum healthcare as low-threshold outpatient clinics, legal restrictions have already been structurally incorporated. This increases the likelihood of successful ACCs while also ensuring additional access control via prior prioritization by healthcare personnel. System development or evolution has served to restore normality, towards a new “normal”. A normality where tensions between political and health related issues [76, 77] manifest in regular implicit or explicit border struggles: Where does medicine end, and politics begin and vice versa? At times, the answer seems not clear, and boundaries appear blurred in this setting. Healthcare for the general population is also permeated by political decisions and social power relations, but in the context of health care for asylum seekers, this proximity is in many ways more apparent in everyday practice.

We observed a coexistence of multiple voices, that have to be brought into “workable arrangements” on a daily basis [45]. Some of these voices are conflicting and explicitly negotiated, while others may potentially be drowned out by this noise or suffocated by bureaucracy. Although the field of asylum seekers' healthcare care can be highly politicized, we think its polycontextural nature may have a de-politicizing effect, discouraging political activism. As actors strive to fulfill their duties, establish routines and communication chains, they are constantly faced with diverse and often conflicting expectations, necessitating internal and external justification of their actions. Health professionals contemplate: Is the treatment truly necessary at this moment? Is it indispensable? How can I justify its necessity to ensure coverage of costs? While government administrators consider: How will I explain rejecting requests despite evident suffering? How can I justify providing treatment independently of asylum logistics? All actors are consistently preoccupied with trying to anticipate and “do right” by communicated and imagined expectations, engaging in struggles over individual cases and local micro-politics at various levels. Since the daily struggle with bureaucracy to meet one's own professional standards and the needs of those being cared for is already perceived as exhausting, there might not be much energy left, to advocate for structural changes on a higher political level. One nurse explained that sometimes they simply cannot or do not do more, since they “are already fighting against so many windmills” (Nurse1P). Is all the bureaucratic effort combined with regularly frustrating experiences that health professionals need to go through to provide proper care to asylum seekers discouraging them from engagement – whether for individual patients or this patient population as a whole – and making them more compliant with administrative rationales? Further research is needed to examine a potential, counter-intuitive depoliticizing effect of working in highly politicized settings that require a continuously high level of personal engagement to stay true to own professional convictions in the face of structural discrimination and inequities.

In the case of restrictive approval and communication practices of local authorities, the mentioned daily struggles or fights happen on an already marked-out field: We observed that many doctors and nurses perceived the status quo as being lower than it actually is if the legal leeway would be exhausted. Why is this the case? There could be several reasons. Firstly, a lack of knowledge about the legal framework. Führer et al. [78] observed an insufficient representation of the scope of benefits that asylum seekers are entitled to in medical literature. Our findings confirm that health professionals are not always familiar with the exact content of the legal provisions for asylum seekers' healthcare. As a result, many health professionals are also unaware that the law is very vaguely formulated. Many believe that they can hardly make a difference in their position and are not fully aware of their powerful role as its interpreters and decision-makers. Therefore, teaching, research, and professional associations must provide training and information, as well as develop guidelines and strategies to strengthen ethical convictions and a self-understanding as health professionals who cannot be instrumentalized for political goals. Related to the aforementioned knowledge gap regarding legal provisions, secondly, professionals might derive consequences for treatment decisions from the reimbursement modalities, even though it is only a jurisdictional attribution for the cost coverage of medical services, which should not affect the rules of medical practice [79]. And thirdly, the organizational procedures implemented around the ACC – while facilitating everyday communication and decision-making processes with financing agencies – reinforce the underlying restrictive rationale of double-checking if something is *really* necessary. Through financing arrangements, policy makers have successfully ensured that migration politics and asylum logistics influence decisions within the health system. In summary, we can assume that organized forms of health care for asylum seekers may have the effect of normalizing this new underlying power structures.

### Reflections on theoretical aspects of a medical sub-system for asylum seekers' healthcare

Structural and legal reasons suggest the existence of a distinct system for asylum seekers' healthcare in Germany. Our empirical data provide additional evidence supporting this assumption. To theoretically discuss what distinguishes this system from the general medical system, we will now look at system function and the binary code.^10^ Regarding its function, asylum medicine is still primarily aiming at restoring damaged health [43], but unlike the general system with the prerequisite, that symptoms must be considered as “acute” or the measures to be taken as “indispensable” for health. The general medical system is furthermore structurally coupled with the economic system of society. One of its tasks in this role is to ensure that people remain able to work or can return to work. This coupling is lacking in the asylum medical (sub-)system, where asylum seekers are mostly not treated so they are able to go to work (again). So a further theoretical question would be: What function does asylum medicine fulfilling for the societal system as a whole? Does it aim to protect the majority population from diseases and/or aim to maintain the minimum requirements of international conventions (like e.g. [80, 81]) and adhere to constitutional provisions for the protection of human dignity?

In addition to the system function, we pointed out in the introduction a distinctive binary code of each system. According to Luhmann, every system relies on a binary code to decide upon its actions or inactions. The medical system conventionally operates based on the distinction of “ill/healthy”, normally reacting actively if it detects “illness” (or potential illness). In specific medical fields, we can observe adjustments in this regard, for example when illness is detected, but considered incurable. This influences treatment decisions, giving rise to the medical sub-system of palliative medicine, which operates under different rationales. However, not every medical specialty can be classified as a sub-system; deviations in system features must be analyzed for each case.

When the code changes, the system changes. Based on our empirical findings about organized care for asylum seekers, we have observed that the medical system has undergone some transformations. In cost coverage negotiations regarding illness or not, the line between this primary distinction becomes frayed. The leading binary code (ill/healthy) in asylum medicine fragments into sub-codes such as “acute/not acute”, “timely treatment needed/postponable”, which now dictate activity or passivity. So the theoretical question is: What do we consider a significant deviation from the original code? If the system, for example, does not act even though an illness has been diagnosed, because it is not considered acute, it has indeed undergone a substantial transformation. Or if we consider it a significant deviation that a medically necessary intervention is not carried out because it is deemed postponable until the patient is transferred elsewhere, deported [54], or granted recognition, then – also from a system theoretical perspective – asylum medicine is (creating) a sub-system of society's medical system.

Does that mean the claim of an existing parallel system for asylum seekers' health care in Germany can be confirmed theoretically? Our data and their theoretical analysis suggest this. But we prefer to speak of a sub- or part-system, not of a parallel system, since this term would not adequately reflect inherent interactions, reference and relationship networks, couplings and therefore mutual dependence between systems. While remaining part of the medical system, the asylum medical sub-system of rationales and communications has some distinctive features, some of which have been described in this article.

### Reflections on polycontexturality and the coupling of differing rationales: implications for equity in health care

The asylum medical sub-system is characterized by polycontexturality. The distinct contextures of law, economics, politics, and medicine are not negated in favor of a singular perspective, such as the legal one; rather they are brought “closer to each other” and are made “more relevant to each other” [45]. Their proximity necessitates explanations. In asylum medicine, different and often conflicting rationales come very close to one another, putting pressure on the actors. They seem to feel the need to explain not only to themselves but also to others when they deviate from their primary code, as well as when they utilize it. For instance, when an application for cost coverage is submitted based on medical necessity, this necessity must be justified and substantiated in accordance with the requirements of the administrative-political system. During the ACC-process the various logics “become more reflexive about their own boundaries and their own identity through the communicative demands of the other logics” [72]. On the one hand, the proximity of government authorities and thus political rationales is identity-forming for the health system and its professionals. On the other hand, there is also significant pressure for flexibility. The same applies in reverse, the administrative personnel also perceive this pressure from the medical side.

Theoretically speaking, in treatment decisions different rationales are dynamically actualized. The respective leading rationale of a system is not being replaced by others but has to interact with them [45]. In the introductory theoretical chapter, it was noted that, in a strictly theoretical sense, the political system is active when power is exercised to enforce migration policies, while the medical system is active when illness is detected and actions are taken to restore health. The significance of this becomes apparent in everyday (moral) negotiations of deservingness and whenever migration-logistical considerations become relevant for health professionals' decision-making. For example, when a healthcare professional does not request funding for a measure that would normally be initiated immediately (if they were to follow medical rationales/norms/justifications), because they assume the patient does not have “proper” reasons for seeking asylum and might soon face deportation. Similarly, when a professional considers a pending transfer of their patient during treatment planning, they actualize the political contexture. Oversimplified,^11^ one could say that in these moments, the physician “becomes part” of the political system. Conversely, whenever administrative employees consider, based on medical data, whether a patient is sick enough to justify treatment, the administrator acts as an agent of the medical system at that moment.

Even if the rationales of other systems are rejected, for example, when an administrator makes a different decision regarding a patient´s health status than suggested by a physician or if a physician refuses to discriminate based on a patient's country of origin, both relate their operations to a previously “foreign” distinction, thereby internalizing it. The organized system of healthcare financing for asylum seekers generates and connects decisions (cf. [82]), linking actors who relate to each other’s expectations and rationales in their decisions in one way or the other, thereby coupling the rationales.

Restrictive, yet vague, legal requirements allow or encourage all actors who are involved in their implementation to intentionally or unintentionally become (restrictive) migration political actors [83, 84], this exacerbates health disparities for asylum seekers in Germany. Therefore, while transparency of processes, criteria and consequences should be pursued, the abolition of the Asylum Seekers Benefits Act, or at least a limitation of its application to the first three instead of 36 months after arrival, should still be advocated for, especially at a time when the duration of the legal restrictions has just recently been prolonged, with expected negative consequences for health, economy and society [85, 86].

## Strength and limitations

Our exploratory ethnographic approach provided insights into previously opaque processes and dynamics related to the implementation of legal restrictions on healthcare provision. By comparing maximally different cases, we were able to reflect on common prioritization criteria of healthcare delivery to asylum seekers and assign these criteria to the logics of functional systems of society. We could develop an ideal-typical flow chart of the cost coverage process as well as understand related systemic transformations.

Our exploratory data was gathered at two field sites, meaning it reflects what was the case at those specified times and locations. For the ideal-typical representation of the cost coverage process, the peculiarities of the processes at both locations were divided from the accounts. It is conceivable that the situation at the described locations may change, for example, the composition of personnel and turnover may change, which can influence processes and once again affect power structures. Additionally, new processes and communications may have been implemented there at a later time.

Even though it is highly likely that our findings show typical features of service delivery processes according to §6 ASBA in many other healthcare facilities for asylum seekers, where electronic health cards are not yet implemented,^12^ they cannot be generalized, as the concrete implementation of the Asylum Seekers Benefits Act varies across the country at the federal state level [66], but also at the level of administrative districts and municipalities. Since the implementation is also based on local negotiation processes, further local and organization-specific variations are to be expected. Still, additional research like the one presented is necessary, to work towards an overarching understanding that reveals micro-politics of power, structural adaptions, and systemic developments, for a more comprehensive understanding of asylum medicine beyond merely stating “it is different everywhere”.

In the frame of this study we also conducted interviews with interpreters, asking them – among other things – for observations regarding the ACC process and prioritizations. However, they were considered as additional informants to provide context and offer an additional observational perspective on these matters to validate our findings. We observed that at times, they get involved and moderate or even influence the decisions, however in the analysis of the present paper, we chose to focus on actors directly involved in process and decision-making, hence we did not include quotes from the translators. In future articles with different focuses, we will also address these valuable insights. Another group of actors is also underrepresented in our study, not because they lack direct responsibility regarding the ACC process but due to challenges in recruitment within the framework of the ethnographic field exploration: The medical consultants. This limitation affects our findings as the information on their background, affiliations, and role is based on second-hand accounts. Initial attempts at contact and engagement proved to be very challenging. This fits in with these actors often being experienced as a black box also by healthcare and government actors. Since the main focus of this project was on the impact of legal restrictions on the health system and its structures, further efforts were not made to recruit some of them after the field phase. A future study should delve deeper into their perspective.

We want to highlight that we observed a high level of commitment and perseverance of healthcare staff, who do their best every day to support patients where they can. We were confronted with fatigue and sometimes resignation, but we also met people on health care and administrative side who were committed to changing local practices and decision-making processes to improve care. One actor also endeavored to inform the public about the challenges of health care for asylum seekers and the need for political action on human rights-based grounds. In this case, frustration – contrary to one of our hypotheses in the discussion – led to political engagement. Our critical reflection on certain aspects of the translation process of legal requirements into the healthcare system should not diminish the great commitment of these actors. Nevertheless, we deem it necessary to point out the side effects of organizational pragmatism regarding the interpretation of the law and its implementation.

Further limitations that need to be addressed pertain to the theoretical analysis. We chose a somewhat unconventional system theoretical approach, because this theory views systems as effective communication structures that follow a specific logic. Looking at that logic or rationale, one can determine which system is currently active, regardless of the organization in which the communication/action/decision takes place. This theoretical approach helped us to assign prioritization criteria to a specific system, without having to assign actors to this or that system. It helped to reveal new interconnections of rationales in asylum medicine pointing to problematic shifts of perspectives of health care personnel. We also could identify indications of a “new” subsystem. However, we applied only a few ideas from Luhmann's extensive theory to assess if we have found more evidence regarding the suspicion that a parallel healthcare system for asylum seekers has emerged in Germany and to describe some of its properties. It can be considered a limitation that we solely pursued this objective and did not endeavor to theoretically encompass all our empirical data within that theoretical framework or further advance the theory.

## Conclusion

Understanding how legal restrictions on asylum seekers' healthcare translate into the healthcare system and how political and medical rationales interweave in this process is important for science and practice actors to navigate this field more consciously. We observed the process of applications for the coverage of medical costs, according to §6 ASBA, for asylum seekers in Germany to investigate the organizational and systemic implications of legal care restrictions. Our findings indicate that implementing care restrictions leads to structural changes within the healthcare system. A cascading prioritization process is introduced, which involves government administrations as financing agencies. It alters tasks, relationships, and power dynamics within this part of the health system. We encountered translatory challenges in reconciling different systems' perspectives and codes.

Requests for cost coverage can have different outcomes depending on a polycontextural assemblage of criteria. The criteria used by health professionals and administrative staff demonstrate a complex interplay of multiple coexisting, sometimes conflicting medical, legal, economic, and political rationales that are dynamically enacted on a daily basis.

Organized forms of healthcare for asylum seekers, such as outpatient clinics in reception centers, act within a highly politicized field but may have de-politicizing effects. They do indeed provide low-threshold access to care, but paradoxically regulate access at the same time, as they have already incorporated restrictive requirements into their structures. They occupy their actors with daily bureaucratic struggles and micro-political negotiations. As a result, many involved actors perceive the legal regulations as stricter than they are, and therefore underestimate their interpretive power. Actors might also be discouraged from political activism, since the daily organizational and sometimes emotional effort is already high and own opportunities to change the existing conditions might be evaluated as being low.

Our exploration of the systemic implications of care restrictions shows that – also from a theoretical perspective – asylum medicine deviates from medicine for the general population in terms of a divergent system function, a higher frequency of shifts in reference system actualizations in decision-making and communication processes, and a fraying of the medical code in the face of the powerful influence of other rationales. By linking organizational systems, migration policy rationales have been effectively integrated into the healthcare system, exacerbating health disparities for asylum seekers in Germany.

## Supplementary Information

Additional file 1. Law text passages relevant to healthcare for asylum seekers.

Additional file 2. Functional systems relevant to health care decisions for asylum seekers.

Additional file 3. Interview participants and pseudonymization.

Additional file 4. Documentation of outcomes of Applications for Cost Coverage (ACCs).

## Data Availability

The ethnographic data used in this publication were obtained through observation. The participants consented to the researcher’s participation and the dissemination of the results in aggregated, condensed form with brief excerpts from interviews and conversations, that are provided within the text. To protect participants’ identities, we cannot share detailed field notes or complete interview transcripts with third parties. Data from the documentation of the ACCs are available upon reasonable request to: SektionEquityMig.Amed@med.uni-heidelberg.de.

## Abbreviations

ACC: Application for cost coverage of medical services
ASBA: Asylum Seekers Benefits Act (in German: Asylbewerberleistungsgesetz)

## Footnotes

1 This period, which specifies how long persons must reside in the federal territory without significant interruption in order to receive social benefits that are similar to those of citizens, has been changed several times in different directions in recent years. Initially, the period was 12 months (1993), then 36 months (1997), 48 months (2007). After the Federal Constitutional Court intervened (in 2012), it was 15 months, then 18 months (2019) – which was the case at the time of this study. It has now been extended again to 36 months (since February 2024).

2 The access barrier can be lifted sooner, if refugee status is granted before the expiration of 36 months. In specific cases a prolonged or extended restriction is possible, for example, in case of non-compliance with the obligation to cooperate with the authorities (e.g., § 15 Abs. 2 Nr. 6 AsylG).

3 “Tolerated stay” means a temporary suspension of deportation is granted. This status is given to people whose asylum applications have been rejected, but they cannot be deported due to various reasons such as unsafe conditions in their home country, refusal of this country to take them back, lack of a passport and inability to obtain one. During this period, the person is allowed to stay in Germany temporarily, but their legal status is uncertain and subject to change based on their circumstances (§ 60a Residence Act).

4 For an English translation of the legal text, see Additional file 1.

5 Functional systems according to Luhmann are: politics, economy, law, science, education, mass-media, love/intimate relations, medicine, sports, art, religion.

6 During the publication process of this article, the waiting period after which the health care restrictions (see introduction and Appendix 1) are lifted was increased from 18 to 36 months.

7 There has been no direct contact to medical consultants in the frame of this study. Therefore, all information on these actors stems from third parties.

8 In the most extreme case, we observed the involvement of: an external specialist, a general practitioner, a nurse, and two other facility doctors (see above: additional procedure of outpatient-clinic “physicians”).

9 At one field site, the documentation sheet of outgoing ACCs and incoming decisions provided by the ethnographer was still used even after the fieldwork to gain an overview of this matter.

10 Polycontexturality as system-feature will be discussed in the following chapter Reflections on polycontexturality and the coupling of differing rationales: implications for equity in health care.

11 This simplification may not align with system theory; however, to state the actors “become” someone or something else underscores the implications of dynamic perspective shifts of actors in practice. For instance, a doctor “becomes” an asylum decision-maker evaluating appropriate reasons for fleeing. Meanwhile, an administrator “becomes” a decision-maker regarding measures that can determine whether an individual patient lives or dies. In the course of the cost coverage process, the actors are apparently being invited to consider matters that do not actually fall within their primary area of expertise. Such “transformations” should be made transparent to facilitate reflection.

12 The same legal restrictions officially apply when using the electronic health card, but the administrative effort for providing benefits is reduced, the number of co-decision makers is reduced and actual access improves.
