# Supplementary material for: Translating restrictive law into practice: An ethnographic exploration of the systemic processing of legally restricted health care access for asylum seekers in Germany
Source: Int J Equity Health. 2024 Oct 10;23:208. doi: 10.1186/s12939-024-02251-y (PMC11465860; doi:10.1186/s12939-024-02251-y)
Supplement: Supplementary file 1 — Additional file 1. Law text passages relevant to healthcare for asylum seekers. [file 12939_2024_2251_MOESM1_ESM.pdf]

Additional file 1. Law text passages relevant to healthcare for asylum seekers

Asylum Seekers Benefits Act (translated from original source: Asylbewerberleistungsgesetz)

§4 Services in case of illness, pregnancy, and childbirth

(1) For the treatment of acute diseases and painful conditions, the necessary medical and dental treatment, including the provision of medicines and bandaging materials, as well as other services required for recovery, improvement, or relief from diseases or their consequences, shall be provided. For the prevention and early detection of illnesses, vaccinations are provided in accordance with §§ 47 and 52 (1) of the Twelfth Book of the Social Code and medically necessary preventive examinations are carried out. Provision of dental prostheses shall only be made where it is medically urgent on a case-by-case basis.

(2) Expectant mothers and women in childbirth shall be provided with medical and nursing care and support, midwifery assistance, medicinal, bandaging, and healing materials.

(3) The competent authority ensures the provision of services under (1) and (2). It also ensures that beneficiaries are offered timely completion of their vaccination protection. Where services are provided by resident physicians or dentists, remuneration is based on contracts applicable at the location of practice of the physician or dentist pursuant to § 72 and § 132e (1) of the Fifth Book of the Social Code. The competent authority determines which contract applies.

§6 Other Benefits

(1) Other benefits can be granted, in particular, if they are indispensable in individual cases to secure the livelihood or health, necessary to meet special needs of children, or required to fulfill an administrative duty to cooperate. The benefits shall be provided as in-kind benefits, and in the presence of special circumstances, as cash benefits.

(2) Persons holding a residence permit according to § 24 (1) of the Residence Act and who have special needs, such as unaccompanied minors or persons who have suffered torture, rape, or other severe forms of psychological, physical, or sexual violence, shall be granted the necessary medical or other assistance.
