# Supplementary material for: Translating restrictive law into practice: An ethnographic exploration of the systemic processing of legally restricted health care access for asylum seekers in Germany
Source: Int J Equity Health. 2024 Oct 10;23:208. doi: 10.1186/s12939-024-02251-y (PMC11465860; doi:10.1186/s12939-024-02251-y)
Supplement: Supplementary file 2 — Additional file 2. Functional systems relevant to health care decisions for asylum seekers. [file 12939_2024_2251_MOESM2_ESM.pdf]

Additional file 2. Functional systems relevant to health care decisions for asylum seekers

(compilated from Künzler, 1990; Luhmann, 2005b; Pelikan, 2007; Roth and Schütz, 2015):

System	Medium	Code	Program[a)]	Function
Medical System	illness	ill/healthy	diagnoses and therapies	"restoration of damaged health"
Legal System	norm	legal/illegal	law	"stabilization of normative expectations"
Economy	money	payment/non-payment	price	provision / distribution
Political System<br>1) Political subsystem (parties)<br>2) Bureaucracy/administration[b)]	power	government/opposition<br>superior/inferior	ideology	power control / limitation

Notes:

a) Copied from Roth/Schütz 2015, S. 24, Luhmann quotes there

b) Function of bureaucratically organised administration: "production of binding problem decisions under the condition of already reduced complexity" (Luhmann 2010, p. 151f).
