# Supplementary material for: Translating restrictive law into practice: An ethnographic exploration of the systemic processing of legally restricted health care access for asylum seekers in Germany
Source: Int J Equity Health. 2024 Oct 10;23:208. doi: 10.1186/s12939-024-02251-y (PMC11465860; doi:10.1186/s12939-024-02251-y)
Supplement: Supplementary file 3 — Additional file 3. Interview participants and pseudonymization. [file 12939_2024_2251_MOESM3_ESM.pdf]

Additional File 3. Interviewee details and pseudonymization

Profession	No.	Gender	Age	Pseudonym[a)]	Site	Time of interview[b)]	Interviewer
physicians	1	w	31-40	Doc1E	E	1	SZ
	2	m	51-60	Doc2E	E	1	SZ
	3	w	31-40	Doc3E	E	2	SZ
	4	m	61-70	Doc1P	P	2	SZ
	5	w	61-70	Doc2P	P	2	SZ
nurses	6	m	41-50	Nurse1I	I	2	SZ
	7	w	51-60	Nurse1E	E	2	SZ
	8	w	41-50	Nurse1P	P	2	SZ
	9	w	51-60	Nurse2P	P	3	SZ
	10	w	41-50	Nurse3P	P	3	SZ
administrators	1	w	21-30	Admin1P	P	2	SZ
	2	w	21-30	Admin2P	P	1	SZ
	3	w	41-50	Admin1P	P	2	SZ
	4	w	31-40	Admin1E	E	1	KB
	5	m	41-50	Admin2E	E	2	KB

Notes:
a) Indicated locations: E = field site 1, P = field site 2, I = explorative interview with external employee in relation to site 1
b) Indicated time of interview: 1 = before field study; 2 = during field study; 3 = after field study
