# Supplementary material for: Translating restrictive law into practice: An ethnographic exploration of the systemic processing of legally restricted health care access for asylum seekers in Germany
Source: Int J Equity Health. 2024 Oct 10;23:208. doi: 10.1186/s12939-024-02251-y (PMC11465860; doi:10.1186/s12939-024-02251-y)
Supplement: Supplementary file 4 — Additional file 4. Documentation of outcomes of Applications for Cost Coverage (ACCs). [file 12939_2024_2251_MOESM4_ESM.pdf]

Additional file 4. Outcomes of requests for cost coverage

For 54 patients 110* requests for services were documented during the field time.

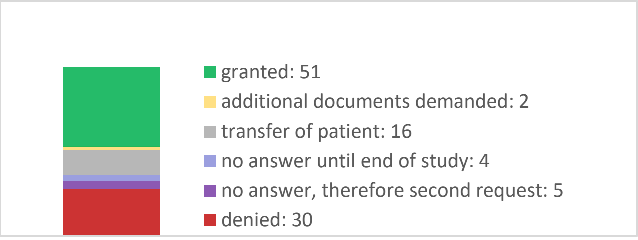

In the clinic of facility 1 (first reception) 38% of 39 decided requests were granted, in facility 2 (shared accommodation) 86% of 42.

*(Missing values: 2, as apart from the application documentation, no further information was available)
